# Survival of compromised adult sensory neurons involves macrovesicular formation

**DOI:** 10.1038/s41420-022-01247-3

**Published:** 2022-11-24

**Authors:** Anand Krishnan, Aparna Areti, Prashanth Komirishetty, Ambika Chandrasekhar, Chu Cheng, Douglas W. Zochodne

**Affiliations:** 1 Neuroscience and Mental Health Institute and Division of Neurology, Department of Medicine, University of Alberta, Edmonton, Alberta Canada; 2 Department of Clinical Neurosciences, University of Calgary, Calgary, Alberta Canada; 3 Department of Anatomy, Physiology, and Pharmacology and Cameco MS Neuroscience Research Centre, College of Medicine, University of Saskatchewan, Saskatoon, Saskatchewan Canada

**Keywords:** Cytoskeleton, Neuroscience

## Abstract

Adult neurons are recognized as post-mitotically arrested cells with limited regenerative potential. Given these restraints, it is perplexing how neurons sustain routine physiological and occasional reparative stress without compromising their density and integrity. We observed that specific insults or physiological alterations drive adult sensory neurons to attempt cell cycle entry. In this context, we demonstrate that at least a small population of sensory neurons modify their cytoskeleton as a survival mechanism in settings of growth arrest and associated stress. Most notably, among their apparent survival modifications is included a unique, and uncharacterized form of macrovesicle shedding and a subsequent neuron size adjustment. Using time-lapse imaging, we demonstrate macrovesicle shedding in some neurons subjected to growth restraint, but not associated with apoptosis. In axotomized neurons in vivo, cell cycle entry was rare to absent and macrovesicles were not observed, but we nonetheless identified changes in mRNA associated with autophagy. In vivo, neighbouring macrophages may have a role in modifying the neuron cytoskeleton after axotomy. Overall, the findings identify previously unrecognized structural adaptations in adult sensory neurons that may provide resilience to diverse insults.

## Facts

- Survival adaptations of post-mitotically arrested sensory neurons against routine stressors in adult organisms are not well understood.
- This study identified a novel adaptation in adult sensory neurons involving macrovesicle formation in response to growth restraint and mitotic stress.
- The macrovesicle-mediated adaptation results in cytoskeletal reprogramming and size reduction of surviving neurons.

## Open question

- Is the survival adaptation part of the natural defence against neurodegenerative conditions?

## Introduction

Adult tissues encounter various forms of mechanical and physiological insult in everyday life. In order to counteract potential damages from such insults, tissues are gifted with intrinsic but varied regeneration potential. For example, adult skin and intestine regenerate faster, whereas regeneration of cardiac and nerve tissues are limited [1]. Adult neurons are post-mitotically arrested cells, which make them incapable to self-renew through proliferation. Therefore, neuron regeneration often refers to the elongation of axons from viable neuron perikarya. Although this is a slower process, this form of repair and recovery is sometimes precluded by neuronal death and loss from major insults. Indeed, the entire neuronal network would be compromised if accumulated neuronal loss occurs.

Since neurons are critical cells that serve basic requirements, there has been a genuine interest in exploring the feasibility of adult neurogenesis. Many studies in this direction have indicated that forced cell cycle entry culminates in neuronal death [2]. Interestingly, adult neurogenesis has been reported in hippocampal neurons [3]. However, in the peripheral nervous system (PNS), such observations are limited. A few studies argue that peripheral neurogenesis is possible through trans-differentiation of satellite glial cells, but additional studies are required to make concluding remarks [4, 5]. Although still in debate, previous studies showed that severe nerve injury secondary to axotomy leads to the loss of peripheral neurons [6]. We previously demonstrated that peripheral nerve injuries trigger DNA damage in sensory neurons potentially risking their integrity [7]. However, we could not find evidence for actual retrograde adult neuronal loss resulting from such injuries, indicating that either the neurons replenish themselves or have unknown natural mechanisms to counteract the severe disruptions encountered during axon damage.

In this study, we found that DRG neurons may enter the cell cycle while responding to extreme growth insults and mitotic stimuli. In light of this, we examined the fate of adult sensory neurons in culture when they were growth compromised. We tracked the surviving neurons and found new adaptive morphological changes in them, enabling their survival. We found that neuronal blebbing and macrovesicular formation as critical events in this adaptation. Overall, this novel study provides insights into previously unrecognized morphological adaptations in neurons that bestow them with survival advantages.

## Results

### Adult sensory neurons attempt to enter cell cycle

**Fig. 1:**
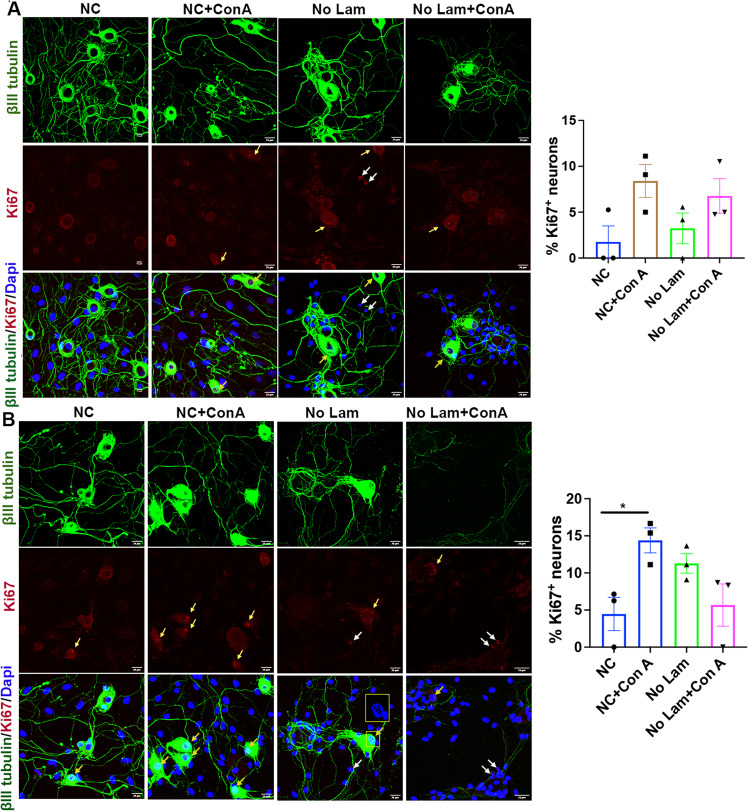
Sensory neurons attempt cell cycle entry. Primary sensory neurons from adult rat DRGs show mild Ki67 expression *(shown by yellow arrows)* in 3-day **A** and 7-day **B** cultures at the indicated experimental conditions suggesting attempt for cell cycle entry. White arrows indicate Ki67 expression in satellite glial cells. Corresponding quantification is shown on the right panel. Results are expressed as percentage of neurons expressing Ki67. The total number of neurons were identified using βIII tubulin staining [data presented as mean ± SEM; *n* = 3; *p ≤ 0.05; ANOVA (Tukey’s multiple comparisons test)].

To understand how adult neurons adapt themselves to survive stress in growth compromised conditions, we cultured the neurons in four different culture conditions: (i) normal control (culture in PLL and laminin coated wells and in 10% FBS/DMEM/F12 media), (ii) normal control + Con A (normal control culture stimulated with the mitogen Con A [8, 9]), (iii) no laminin culture (normal control culture without laminin coating to restrict outgrowth), (iv) no laminin + Con A (normal control culture without laminin coating but Con A stimulated). The culture conditions ii–iv provide either mitotic stress (culture ii) or growth restriction due to laminin deprivation (culture iii), or both (culture iv). We did not supplement NGF in these cultures to further diminish their natural outgrowth. Interestingly, all culture conditions induced mild expression of Ki67 (marker for cell cycle) in neurons, with culture conditions ii-iv showing more expression in both 3 and 7 day cultures indicating increased attempt of neurons for cell cycle entry under growth restricted and stressed conditions (Fig. 1A, B).

**Fig. 2:**
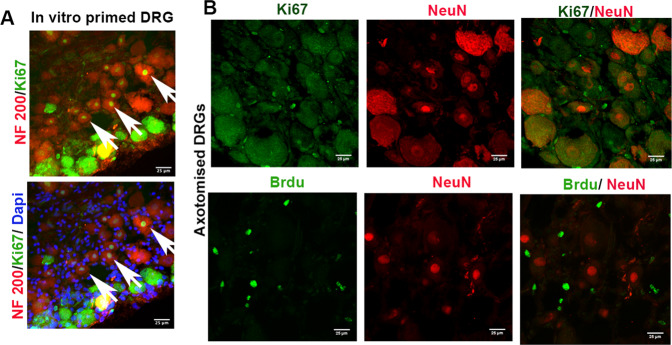
Sensory neurons attempt cell cycle entry. **A** Sections of adult rat DRGs incubated in vitro for three days *(*in vitro *priming)* in DMEM/F12/FBS media show Ki67 expression in neurons. Arrows indicate nuclear expression of Ki67 and its co-localization with the nuclear stain dapi [scale bar, 25 µm]. **B** Sections of DRGs from sciatic nerve injured *(transection injury)* adult rats do not show Ki67 expression or Brdu incorporation in neurons after 3 days of injury, evident by lack of co-staining of NeuN with Ki67 or Brdu [scale bar, 25 µm].

We then confirmed the potential of neurons to enter the cell cycle through an additional approach. We have recently demonstrated that sensory neurons from in vitro primed *DRGs (done by incubating whole DRGs* in vitro *for 24* *h)* show enhanced outgrowth [10]. However, we noted that extended in vitro incubation times of 48-72 h are stressful for neurons leading to neuron loss. Hence, we performed an extended in vitro incubation of DRGs to investigate neuron behavior. We found that in vitro priming for 72 h induced Ki67 expression at least in some neurons, indicating that severe stress signals may drive adult neurons to attempt cell cycle entry (Fig. 2A). To compare this artificial stress paradigm with an injury-driven stress condition, we checked the possibility of cell cycle entry in axotomized neurons three days after complete transection injuries to sciatic nerve. However, we did not find neuronal expression of Ki67 in axotomized DRGs in vivo. Similarly, there was no BrDU uptake (an assay to detect S phase activity) in axotomized DRGs within three days of axotomy (Fig. 2B). Taken together, our in vitro observations indicate that adult neurons have a potential to enter the cell cycle at stressed conditions, challenging the conventional understanding that adult neurons are strictly post-mitotically arrested cells. However, efficient regulatory mechanisms may be operational in vivo preventing the neurons from entering the cell cycle even at severe stressful conditions, and thereby preserve the integrity of neuronal network.

### Forced cell cycle entry of sensory neurons induces abnormal distribution of the neuronal nuclear marker NeuN

**Fig. 3:**
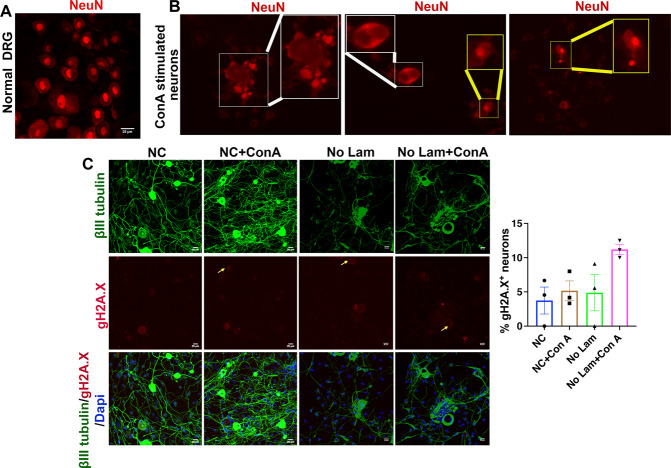
Distribution of NeuN and expression of gH2A.X in stressed sensory neurons. **A** Distribution of NeuN in a normal DRG. **B** NeuN distribution in ConA stimulated adult sensory neurons. The neurons were cultured in the presence of ConA for 48 h in PLL coated but laminin devoid surfaces. Abnormal distribution of NeuN in large diameter neurons is shown in white boxes while stable nuclear distribution of NeuN in small diameter neurons is shown in yellow boxes. Insets show enlarged views. **C** Expression of the DNA damage marker gH2A.X (yellow arrow) in 7-day cultures at the indicated experimental conditions. The right panel shows the quantification and suggest no major induction of DNA damage in laminin deprived or ConA stimulated cultures compared to control, while an increased trend for DNA damage was observed in No Lam+ConA cultures [data presented as mean ± SEM; *n* = 3; ANOVA (Tukey’s multiple comparisons test)].

Mammalian cell division includes DNA synthesis and mitosis, both of which progress through evident changes in nuclear architecture. Therefore, we examined morphological changes in neuronal DNA after forcing the neurons to enter the cell cycle by mitogen treatments. The neurons were cultured in PLL coated surfaces without laminin and then stimulated with the mitogen ConA in FBS enriched media. The neurons were then stained for NeuN, which specifically marks the neuronal nuclei. We observed abnormal distribution of NeuN in these neurons, especially large diameter neurons, indicating dispersal of its usual localization in nuclei (Fig. 3A, B). NeuN immunoreactivity was scattered diffusely or in granular appearance within neurons, suggesting abnormal distribution or rearrangement. Strikingly and in contrast, the small diameter neurons showed intact NeuN nuclear localization. These observations indicated that either small diameter neurons are less susceptible to exogenous mitogenic stimuli, or they are resilient to coping stress through counteracting mechanisms.

Further, we examined the expression of the DNA damage marker gH2A.X in different culture conditions (culture conditions i–iv; Fig. 3C). We did not find any major induction of gH2A.X in ConA stimulated or laminin deprived cultures compared to control, indicating that, although neurons perceive mitotic demands and stress, evident by Ki67 expression and NeuN distribution, they are resistant to major DNA damage. The βIII tubulin staining was also intact in the neurons showing that they are healthy and suggests that unknown survival mechanisms are active in these neurons to counteract forced cell cycle entry. We, however, noted an increased trend for gH2A.X positivity in No Lam+Con A cultures, suggesting that compounding insults may initiate DNA damage in adult neurons.

### Cytoskeletal reprogramming of neurons involves macrovesicular formation

**Fig. 4:**
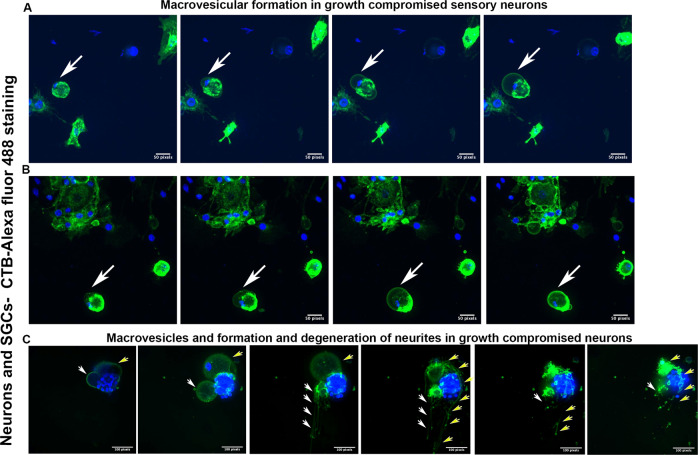
Macrovesicular formation and unstable neurite formation in growth compromised neurons. **A**, **B** Snapshots of the videos (Video 1 and the Supplementary Video 1) generated from overnight time-lapse confocal images of primary sensory neurons grown on PLL coated, but laminin deprived surfaces show macrovesicular formation (arrows). The neurons were stained with Cholera toxin-B conjugated with Alexa fluor-488 (green) and Hoechst (nuclear stain: blue) for the live cell imaging. **C** Snapshots of Video 2 generated from similar cultures as in **A** and **B** show unstable neurites emerging from the macrovesicular structures followed by rearrangement of neuronal cytoskeleton. Two events of macrovesicular formation and fates are shown using white and yellow arrows. [Bar calibration is 2.24 pixels/micron].

Next, we wanted to understand the fate of neurons cultured in outgrowth compromised conditions for longer intervals such as a week. For this, we cultured primary sensory neurons in PLL coated but laminin deprived surfaces and NGF lacking but FBS enriched media. We anticipated that these neurons would either enter the cell cycle or enter apoptosis due to the continued inhibition of their normal physiology. In order to track the neuron’s response, we performed live cell imaging of the cultures on the 4^th^ day. We captured large vesicles formed in populations of these neurons when imaged over 12 h from the imaging onset (Fig. 4A, B and Video 1, and Supplementary Video 1). Macrovesicle formation and shedding, while largely cytoplasmic, was also associated with significant rearrangement of the neuronal nuclei, compatible with our previous observations of NeuN redistribution in growth compromised neurons. The vesicles, after reaching a peak size, either were shed or resolved without neuron duplication, indicating that this cytoskeletal rearrangement may be critical for the survival and maintenance of neurons when their natural growth is compromised. Surprisingly, we also observed that in some instances, neurons attempted to form unstable neurite outgrowths closely associated with macrovesicles, before re-entering into a steady state and resuming a normal configuration (Fig. 4C and Video 2). The overall findings indicated that neurons modify their cytoskeleton through macrovesicular formation for maintaining steady state when natural growth is compromised.

### Small neurons have higher survival potential when natural growth is compromised

**Fig. 5:**
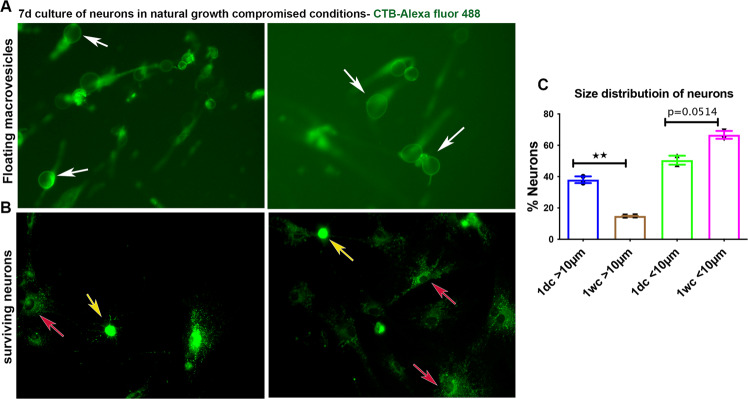
Small diameter neurons survive when natural growth is compromised. **A** Cultures of sensory neurons in growth compromised conditions for one week (PLL coated but laminin deprived growth surface) develop macrovesicles (arrows). The neurons are stained using CTB-Alexa fluor 488 for the visualization of macrovesicles. **B** One week culture of sensory neurons in growth compromised conditions demonstrates that small diameter neurons have comparatively higher survival rate (yellow arrows). Red arrows indicate glial cells. **C** Quantification of large (>10 µm) and small (<10 µm) dimeter neurons in one-day (1d) and one-week (1w) neuron cultures in growth compromised media shows reduced presence of large diameter neurons and increased presence of small diameter neurons in one-week cultures compared to one-day cultures [data presented as mean ± SEM; *n* = 2; **p ≤ 0.01; Student’s t test].

We found numerous floating macrovesicles in neuronal cultures grown on outgrowth restricted surfaces, indicating frank shedding of these structures (Fig. 5A). Since the macrovesicles we observed were generated from the neuronal cytoskeleton, we wanted to examine if such a modification alters the size of surviving neurons. In order to examine this, we quantified the size distribution of neurons in one day and one-week cultures separately after seeding equal number neurons in both these cultures. A cut off diameter of >10 µm and <10 µm were considered as large and small dimeter neurons, respectively. Strikingly, there was a significant reduction in the percentage of large diameter neurons in one-week cultures while the percentage of small diameter neurons increased in these cultures (Fig. 5B, C). This observation suggest that large diameter neurons may modify their structure through extensive macrovesicular formation for survival and to acquire more stable small diameter phenotypes.

### Macrovesicular shedding in vivo is undetected: Does resident macrophage contribute to neuron cytoskeletal remodelling?

**Fig. 6:**
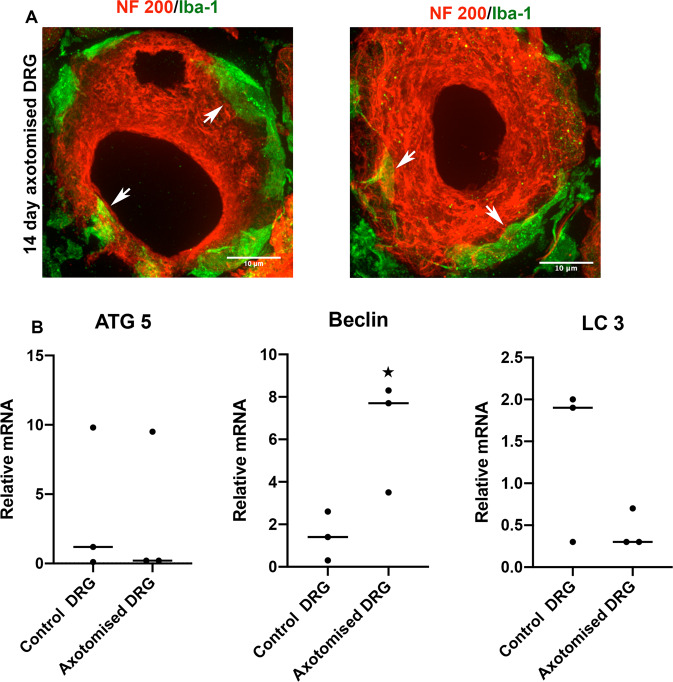
Macrophages and autophagy may involve with neuronal cytoskeletal rearrangement. **A** Confocal images of immunostaining of Iba-1 (macrophages-green) and NF200 (neurons-red) in DRGs after 14 days of sciatic nerve axotomy shows intimate contact of macrophages with the neuronal cytoskeleton (arrows), suggesting that macrophages may involve with the structural re-organization of injured neurons [scale bar, 10 µm]. **B** Quantification of mRNAs involved with autophagy in 3 day axotomized DRGs show significant induction of Beclin, suggesting that autophagy response occurs in DRGs even at an early interval of post axotomy. Individual values and median are shown. *p < 0.05.

We next examined if macrovesicles are developed in DRGs in vivo after axotomy, the most common form of a neuron stress response. However, we could not detect macrovesicles in the DRGs at 3, 7, and 14 days after axotomy. Floating macrovesicles may not be evident in standard tissue sections, but incipient macrovesicle shedding did not appear to be visible in axotomy-stressed neurons. Their absence may indicate resistance to this route of neuron survival and response. However, we wondered if there might be other mechanisms that orchestrate macrovesicle shedding in vivo. Therefore, we searched for cellular and molecular events that could potentially be involved with the formation of macrovesicles. Macrophages are resident immune cells in the DRGs. They increase their turnover after axotomy and perform phagocytosis of axonal debris from damaged nerves. However, their phagocytic activity in DRGs has not been extensively studied. The exceptional dynamics of ganglionic macrophages has been reported recently by our laboratory in separate work [11]. We found evidence that DRG resident macrophages make intimate contact with neuronal cytoskeleton after axotomy. Although speculative, it may be that this intimate relationship sculps macrovesicles from neurons before they are evident (Fig. 6A). The absence of macrophages in our in vitro cultures may have prevented identifying this potential event. We also found changes in autophagy related mRNA, especially changes in expression of Beclin, in axotomized DRGs supporting the concept that these neurons are under stress after axotomy (Fig. 6B). Autophagy is a programmed cellular event involving cytoskeletal components and is associated with cellular survival. Overall, although detailed further investigations are warranted, our study suggests that macrovesicle-dependent restructuring of neurons is a potential mechanism of survival for stressed and compromised adult sensory neurons.

## Discussion

Accumulating literature supports the rule that adult neurons are mitotically incompetent. However, convincing demonstration of the turnover of adult hippocampal neurons has generated interest in adult neurogenesis and found that repopulation of adult CNS neurons involves neural progenitor/stem cell reserve [1]. Such observations are limited in the PNS, however, there were suggestions that damaged adult neurons are occasionally replaced by neuronal trans-differentiation of satellite glial cells (SGCs) [4, 5]. We previously examined this possibility by tracking BrdU incorporated cells in DRGs after peripheral nerve injury. BrdU is a synthetic thymidine analog that incorporates into the DNA during replication, and once incorporated, is retained in the cells. We noted that DRG resident SGCs and macrophages proliferate in response to nerve injuries and incorporate BrdU. However, there was not enough emergence of BrdU positive neurons to verify the trans-differentiation hypothesis at least at the early intervals of post nerve injury [11]. Nonetheless, we previously found basal and injury driven expression of cell cycle inhibitors in sensory neurons, indicating the presence of natural molecular brakes in neurons to prevent them from entering the cell cycle [12–15].

Our experimental approach in this study utilizes an artificial model forcing cell cycle entry in adult neurons but bears significance in understanding how neurons protect themselves from physiological and reparative stress. For instance, nerve injuries upregulate growth factors in the DRGs [16–18]. Some of these growth factors are mitogenic and they induce local proliferation of SGCs and macrophages in the DRGs [11]. These growth factors might trigger neurons to enter cell division, as observed in this study and by others [4, 19]. However, since neurons are unable to successfully complete the cell cycle due to their post-mitotically arrested state, there may be intrinsic safeguard measures to protect their viability when they enter this unconventional route. Our time-lapse experiments in this study agree with this argument and showed that neurons undergo massive cytoskeletal changes to apparently regain their normal state, albeit at smaller caliber.

Previous studies indicate that neurons that attempt cell division or DNA replication eventually die [2, 20]. However, our time-lapse imaging showed that they undergo morphological changes, which may potentially enable their survival. For example, we found that neurons release macrovesicles and reduce their size to accommodate cytoskeletal modifications and maintain survival. There are several points to argue that these macrovesicles may not be involved with induction of apoptosis: (i) apoptosis in general involves the formation of membrane bound small apoptotic bodies, not macrovesicles, (ii) although apoptotic bodies may release in the extracellular environment, their size generally range between 1 and 5 µm. However, the size of macrovesicles induced in our experimental conditions is larger than apoptotic bodies, (iii) apoptotic body formation generally associate with DNA fragmentation. However, although, there was DNA condensation (Dapi: Fig. 4 and videos 1-2, and Supplementary Video 1), DNA fragmentation was absent in our experimental conditions. These observations indicate that the process going on in the neurons in our experimental conditions may not be apoptosis.

Although we observed size reduction in neurons, how this size reduction enables their long-term survival is still a question. Loss of the neuronal cytoskeletal material, neurofilament, without loss of neurons has been reported previously in brain injury [21]. This, along with our observation, indicates that the survival adaptability of neurons precedes cytoskeletal rearrangement. Nonetheless, it is puzzling to understand how a neuron that attempts DNA replication survives in the long term, anticipating that severe DNA damage may occur in the event of an unsuccessful replication attempt. We reported earlier that peripheral nerve injury induces the DNA repair protein BRCA1 in sensory neurons as a protective measure [7]. Therefore, timely repair of DNA by repair molecules may offer protection in this circumstance. One caveat in this study is that we did not examine whether the neurons that undergo size reduction are the same that attempt DNA replication. Further studies focussing on the differential fates, if any, of neurons that attempt DNA replication and size modification may solve this puzzle. This would require longer term live neuron imaging.

We speculate that autophagy and intervention by macrophages may play critical roles in the cytoskeletal adaptations of injured neurons. Autophagy has been known as a survival mechanism in neurons, and defects in autophagy lead to neuronal abnormalities [22]. Autophagy is associated with significant changes in cellular cytoskeleton, and we found changes in autophagy-related mRNA in axotomized DRGs, indicating that autophagy may be operational in these neurons [23]. While autophagosomes are reported to acquire an upper size limit up to 900 nm, the macrovesicles identified in our study is larger, and hence, it would be ideal to call these structures as macrovesicles [24]. On the other hand, macrophages are resident phagocytotic cells in the DRGs and are known to phagocytose extracellular vesicles, including exosomes released by sensory neurons [25]. Here, we found that macrophages wrap around axotomized DRG neurons and make intimate contact with the neuronal cytoskeleton. We have also previously reported that fluorochromes applied to distant axotomized axons undergo retrograde transport to perikarya but are also distributed to perineuronal cells in DRGs [26]. The macrophages may thus help to eliminate survival-directed neuron blebs to support the cytoskeletal modification of injured neurons, facilitating their survival. This indeed throws light into the potential role of DRG resident macrophages in supporting neuron homeostasis in emergency situations.

Overall, our study reveals previously unrecognized cytoskeletal adaptations in growth compromised neurons supporting their long-term survival. We did not examine which specific neuronal subtypes are more susceptible to our experimental challenge and bestowed with the survival advantage presented here. More detailed studies are required to understand the subtype-specific survival and proliferative competences of adult sensory neurons.

## Methods

### In vitro neuron cultures and mitogen treatments

Primary sensory neurons were cultured from dorsal root ganglia (DRG) isolated from adult male SD rats. Briefly, the DRGs were incubated in 0.1% collagenase at 37 °C for 90 minutes for the enzymatic dissociation of individual neurons. This was followed by mechanical dissociation of individual neurons from the DRGs performed by repeated pipetting of the DRGs in suspension. The resulted individual cell suspension was then overlaid on a 15% BSA solution to eliminate the debris at the interface. Finally, the cell suspension was seeded on 4-well chambered slides.

For forced cell cycle entry and DNA damage assessment, the neuron cultures were divided into 4 groups. The groups were, (a) Normal cell culture: *NC* (cultured in Poly-L-Lysine (PLL) and laminin coated wells), (b) NC supplemented with the mitogen Concavalin A (ConA: 0.5 µg/ml): *NC* + *ConA*, (c) Neurons cultured in PLL-coated but laminin deprived wells (for restricting neurite outgrowth): *No Lam*, (d) No Lam cultures supplemented with Con A (0.5 µg/ml): *No Lam* + *ConA*. The neurons were cultured for varying time intervals. For immunocytochemistry, the cells were fixed in 4% paraformaldehyde and blocked and permeabilized in PBS containing 5% donkey serum and 0.3% TritonX100. Ki67 and gH2AX staining were done to assess cell cycle entry and DNA damage in these neurons. The primary antibodies used were rabbit polyclonal anti-Ki67 (Invitrogen, Cat No. PA5-16), mouse monoclonal gH2A.X antibody (Millipore Sigma, Cat No. 05-636-I), mouse monoclonal anti-βIII tubulin antibody (1:600, Sigma Cat No. T8328) and rabbit monoclonal anti-βIII tubulin antibody (1:600, Sigma Cat No. T3526). Cells were incubated with the primary antibodies for 2 h and then washed with PBS. The corresponding secondary antibodies, either tagged with Alexa fluor 488 or Alexa fluor 546 (Invitrogen, USA), were then used for 90 min at room temperature. Sections were washed and mounted in mounting medium containing DAPI to stain the nuclei (Vectashield, Vector Laboratories, USA). The Ki67 & gH2AX positive neurons were counted at random fields (a minimum 10 neurons per replica was considered: total three replicas) using confocal microscopy images. The % number of positive neurons is then calculated relative to total number of neurons and the average values are presented in graphical format.

For size distribution analysis, primary neurons were cultured in 10% FBS and antibiotic/antimycotic containing DMEM/F12 in PLL coated but laminin deprived surfaces. For quantification of neurons based on size cut off, NF200 stained cultures were imaged at random fields at 24 h and 7 day. Counting of neurons was done manually after applying standard scale measurements on the images.

### In vitro priming of dorsal root ganglia

For in vitro priming, whole DRGs were incubated at 37 °C in 10% FBS and antibiotic/antimycotic containing DMEM/F12 media. After 72 h incubation, the DRGs were fixed in Zamponi’s buffer overnight, followed by another overnight incubation in 20% sucrose. Tissue blocks were then made using OCT compound. 12 µm cryosections were used for immunohistochemistry.

### Nerve injury model *(*in vivo *priming or pre-conditioning injury)*, immunohistochemistry and BrdU incorporation assay

Adult male SD rats were used for the study. The Animal ethics committees of the University of Calgary and the University of Alberta reviewed and approved the animal procedures performed in this study. Animals were randomly assigned to the injury and healthy control groups. For the nerve transection injury or axotomy, the sciatic nerve of the animals was exposed at the mid-thigh level, and a complete transection performed under aseptic conditions. The animals were then allowed to survive for 3-14 days before harvesting the lumbar DRGs for immunohistochemical analysis.

For immunohistochemistry, the tissue sections were blocked and permeabilized using PBS containing 5% donkey serum and 0.3% TritonX100. The primary antibodies used were Ki67 (rabbit, Thermo Fisher Scientific, Cat No. PA5-16785), NF200 (mouse, Millipore Sigma, Cat No. MAB5266), NeuN (mouse, Millipore Sigma, Cat No. MAB377), and Iba1 (rabbit; Wako Chemicals, Cat No. 019-19741). The sections were incubated with primary antibodies for 1 hour at RT, followed by incubation with secondary antibodies for another hour. The secondary antibodies used were antimouse-cy3 (Sigma-Aldrich, MO) and antirabbit-Alexa Fluor 488 (Life Technologies, CA). The processed sections were finally mounted using Vectashield mounting medium containing Dapi for nuclear staining.

For the BrdU incorporation assay, 5-Bromo-2´-deoxyuridine (BrdU) (Sigma, MO) was dissolved in 1 mM Tris, 0.8% NaCl, 0.25 mM EDTA solution and made up with phosphate buffered saline (PBS). A dose of 100 mg/kg of BrdU was administered to animals intraperitoneally for four days (including the day axotomy was performed) prior to harvesting the DRGs. For the tissue staining of BrdU, the tissue sections were incubated with 2 N HCl for 30 minutes, washed three times in PBS, and then dipped in 0.01% trypsin at 37 °C for 3 minutes. The tissues were then blocked using 1% BSA containing 0.3% Triton X100 and incubated with BrdU antibody (Rabbit, Thermo Fisher Scientific, Cat No. PA5-32256**)** and NeuN for 1 hour at RT, followed by corresponding secondary antibodies for another one hour at RT. The sections were finally mounted using Dapi containing Vectashield mounting media.

### Real-Time PCR

RNA isolation was done using Trizol reagent (Applied biosystems, CA) as per the manufacturer’s instructions. One microgram of total RNA was converted into cDNA using a cDNA reverse transcription kit (Applied Biosystems, CA). The primers were designed using Primer Express 2.0 software (Applied Biosystems, CA), and reactions were set using SYBR®Green master mix (Applied Biosystems, CA). The comparative CT method (2-ΔΔCT) was then used for quantifying the relative expression of mRNAs. All mRNAs of interest were normalized to the expression of housekeeping mRNAs. The primer sequences are provided in Supplementary Table 1.

### Time-lapse imaging

For the time-lapse imaging of live cells, neurons were live stained using Cholera toxin B conjugated with Alexa fluor-488 (CTB-Alexa 488) and Hoechst. Both CTB-Alexa 488 and Hoechst were added to the culture for 15 minutes at 37 C. The cells were then washed, and new media supplemented. The cells were then imaged overnight using Wave Fx2 confocal microscope (Quorum technologies, ON). Velocity software was used for processing the time-lapse images into the video format.

### Statistical analysis

Results are represented as mean ± SEM. The student’s t-test or One-way ANOVA followed by post-hoc Turkey’s Multiple Comparison Test was performed using GraphPad Prism software. The significance was established with a p-value of ≤0.05. Wherever statistics is applied, the number of biological replicates used (n) is given in the corresponding figure legends. As such, ‘n’ also represents the number of times the experiment was repeated. The mRNA analysis of tissues from the injured and healthy control animals was done in a non-blinded manner.

## Supplementary information

Authorship change agreement

Video 1

Video 2

Supplementary Video 1

Supplementary Material

Supplementary Video 2 legend

## Data Availability

The data supporting the conclusion of the findings are provided in the article file. Additional details are available upon reasonable request.
