# Supplementary material for: Survival of compromised adult sensory neurons involves macrovesicular formation
Source: Cell Death Discov. 2022 Nov 24;8:462. doi: 10.1038/s41420-022-01247-3 (PMC9691713; doi:10.1038/s41420-022-01247-3)
Supplement: Supplementary file 1 — Authorship change agreement [file 41420_2022_1247_MOESM1_ESM.pdf]

# Re: URGENT final approval of Ms and authorship

Krishnan, Anand <anand.krishnan@usask.ca>
Sat 10/29/2022 3:48 PM

To: Douglas Zochodne <zochodne@ualberta.ca>;Aparna Areti <areti@ualberta.ca>;Prashanth Komirishetty <komirish@ualberta.ca>;Ambika Chandrasekhar <ambika2@ualberta.ca>;chu cheng <cheng.chu01@gmail.com>

Thanks Doug.

Please find my approval of the manuscript and the authors' order as listed!

Anand Krishnan

---

**From:** Douglas Zochodne <zochodne@ualberta.ca>
**Sent:** Saturday, October 29, 2022 2:33 PM
**To:** Krishnan, Anand <anand.krishnan@usask.ca>; Aparna Areti <areti@ualberta.ca>; Prashanth Komirishetty <komirish@ualberta.ca>; Ambika Chandrasekhar <ambika2@ualberta.ca>; chu cheng <cheng.chu01@gmail.com>
**Subject:** URGENT final approval of Ms and authorship

CAUTION: External to USask. Verify sender and use caution with links and attachments. Forward suspicious emails to phishing@usask.ca

Dear all
Please find attached the penultimate version of this Ms.
Anand has put all together carefully.
**WE NEED EACH OF YOU TO REPLY BY EMAIL ASAP THAT YOU APPROVE OF THE MANUSCRIPT AND IN PARTICULAR THE AUTHORSHIP ORDER AS LISTED.**
Please respond if able within 48h thanks!
I very much appreciate the long and hard work that has gone into this paper, almost published now!
Thank you.
Dz

--
Douglas W. Zochodne MD, FRCPC
Neurology
Director, Neuroscience and Mental Health Institute
University of Alberta
zochodne@ualberta.ca
780-248-1928 (phone)
780-248-1807 (fax)

## Re: URGENT final approval of Ms and authorship

Ambika Chandrasekhar <ambika2@ualberta.ca>
Sun 10/30/2022 9:21 AM

To: Douglas Zochodne <zochodne@ualberta.ca>

Cc: Krishnan, Anand <anand.krishnan@usask.ca>;Aparna Areti <areti@ualberta.ca>;Prashanth Komirishetty <komirish@ualberta.ca>;chu cheng <cheng.chu01@gmail.com>

CAUTION: External to USask. Verify sender and use caution with links and attachments. Forward suspicious emails to phishing@usask.ca

Hi Dr. Zochodne,
I am ok with the order list.
Thanks
Ambika

On Saturday, October 29, 2022, Douglas Zochodne <zochodne@ualberta.ca> wrote:

> Dear all
> Please find attached the penultimate version of this Ms.
> Anand has put all together carefully.
> **WE NEED EACH OF YOU TO REPLY BY EMAIL ASAP THAT YOU APPROVE OF THE MANUSCRIPT AND IN PARTICULAR THE AUTHORSHIP ORDER AS LISTED.**
> Please respond if able within 48h thanks!
> I very much appreciate the long and hard work that has gone into this paper, almost published now! Thank you.
> Dz
>
> --
> Douglas W. Zochodne MD, FRCPC
> Neurology
> Director, Neuroscience and Mental Health Institute
> University of Alberta
> zochodne@ualberta.ca
> 780-248-1928 (phone)
> 780-248-1807 (fax)

# Re: URGENT final approval of Ms and authorship

Prashanth Komirishetty <komirish@ualberta.ca>
Sat 10/29/2022 8:55 PM

To: Krishnan, Anand <anand.krishnan@usask.ca>

Cc: Douglas Zochodne <zochodne@ualberta.ca>;Aparna Areti <areti@ualberta.ca>;Ambika Chandrasekhar <ambika2@ualberta.ca>;chu cheng <cheng.chu01@gmail.com>

Hi Dr.Zochodne,
I am okay with the authors list.
Thank you.

On Sat., Oct. 29, 2022, 3:48 p.m. Krishnan, Anand, <anand.krishnan@usask.ca> wrote:

> Thanks Doug.
>
> Please find my approval of the manuscript and the authors' order as listed!
>
> Anand Krishnan
>
> ---
>
> **From:** Douglas Zochodne <zochodne@ualberta.ca>
> **Sent:** Saturday, October 29, 2022 2:33 PM
> **To:** Krishnan, Anand <anand.krishnan@usask.ca>; Aparna Areti <areti@ualberta.ca>; Prashanth Komirishetty <komirish@ualberta.ca>; Ambika Chandrasekhar <ambika2@ualberta.ca>; chu cheng <cheng.chu01@gmail.com>
> **Subject:** URGENT final approval of Ms and authorship
>
> CAUTION: External to USask. Verify sender and use caution with links and attachments. Forward suspicious emails to phishing@usask.ca
>
> Dear all
> Please find attached the penultimate version of this Ms.
> Anand has put all together carefully.
> **WE NEED EACH OF YOU TO REPLY BY EMAIL ASAP THAT YOU APPROVE OF THE MANUSCRIPT AND IN PARTICULAR THE AUTHORSHIP ORDER AS LISTED.**
> Please respond if able within 48h thanks!
> I very much appreciate the long and hard work that has gone into this paper, almost published now! Thank you.
> Dz
>
> --
> Douglas W. Zochodne MD, FRCPC
> Neurology
> Director, Neuroscience and Mental Health Institute
> University of Alberta
> zochodne@ualberta.ca
> 780-248-1928 (phone)
> 780-248-1807 (fax)

# Fwd: URGENT final approval of Ms and authorship

Douglas Zochodne <zochodne@ualberta.ca>
Tue 11/1/2022 1:43 PM
To: Krishnan, Anand <anand.krishnan@usask.ca>

CAUTION: External to USask. Verify sender and use caution with links and attachments. Forward suspicious emails to phishing@usask.ca

---------- Forwarded message ---------
From: **Aparna Areti** <areti@ualberta.ca>
Date: Sat, Oct 29, 2022 at 7:12 PM
Subject: Re: URGENT final approval of Ms and authorship
To: Douglas Zochodne <zochodne@ualberta.ca>

Hi Dr. Zochodne,
I am okay with the authorship.
Thanks for making me the part of the paper and the support.
Thank you.

On Sat, 29 Oct, 2022, 2:33 PM Douglas Zochodne, <zochodne@ualberta.ca> wrote:

> Dear all
> Please find attached the penultimate version of this Ms.
> Anand has put all together carefully.
> **WE NEED EACH OF YOU TO REPLY BY EMAIL ASAP THAT YOU APPROVE OF THE MANUSCRIPT AND IN PARTICULAR THE AUTHORSHIP ORDER AS LISTED.**
> Please respond if able within 48h thanks!
> I very much appreciate the long and hard work that has gone into this paper, almost published now! Thank you.
> Dz
>
> --
> Douglas W. Zochodne MD, FRCPC
> Neurology
> Director, Neuroscience and Mental Health Institute
> University of Alberta
> zochodne@ualberta.ca
> 780-248-1928 (phone)
> 780-248-1807 (fax)

--
Douglas W. Zochodne MD, FRCPC
Neurology
Director, Neuroscience and Mental Health Institute
University of Alberta
zochodne@ualberta.ca

# Fwd: URGENT final approval of Ms and authorship

Douglas Zochodne <zochodne@ualberta.ca>
Tue 11/1/2022 1:33 PM
To: Krishnan, Anand <anand.krishnan@usask.ca>

CAUTION: External to USask. Verify sender and use caution with links and attachments. Forward suspicious emails to phishing@usask.ca

[redacted]

---------- Forwarded message ---------
From: **chu cheng** <cheng.chu01@gmail.com>
Date: Tue, Nov 1, 2022 at 1:30 PM
Subject: Re: URGENT final approval of Ms and authorship
To: Douglas Zochodne <zochodne@ualberta.ca>

Dear Dr. Zochone
I am OK with this. Thank you very much.

Chu Cheng

Douglas Zochodne <zochodne@ualberta.ca>于2022年11月1日 周二11:17写道：

> Hi Chu- Are you able to let us know about this today?
> D
>
> ---------- Forwarded message ---------
> From: **Douglas Zochodne** <zochodne@ualberta.ca>
> Date: Sat, Oct 29, 2022 at 2:33 PM
> Subject: URGENT final approval of Ms and authorship
> To: Krishnan, Anand <anand.krishnan@usask.ca>, Aparna Areti <areti@ualberta.ca>, Prashanth Komirishetty <komirish@ualberta.ca>, Ambika Chandrasekhar <ambika2@ualberta.ca>, chu cheng <cheng.chu01@gmail.com>
>
> Dear all
> Please find attached the penultimate version of this Ms.
> Anand has put all together carefully.
> **WE NEED EACH OF YOU TO REPLY BY EMAIL ASAP THAT YOU APPROVE OF THE MANUSCRIPT AND IN PARTICULAR THE AUTHORSHIP ORDER AS LISTED.**
> Please respond if able within 48h thanks!
> I very much appreciate the long and hard work that has gone into this paper, almost published now! Thank you.
> Dz
>
> --
