# Supplementary material for: Survival of compromised adult sensory neurons involves macrovesicular formation
Source: Cell Death Discov. 2022 Nov 24;8:462. doi: 10.1038/s41420-022-01247-3 (PMC9691713; doi:10.1038/s41420-022-01247-3)
Supplement: Supplementary file 5 — Supplementary Material [file 41420_2022_1247_MOESM5_ESM.docx]

**SUPPLEMENTARY MATERIAL**

**Survival of compromised adult sensory neurons involves macrovesicular formation**

Anand Krishnan^1-3^, Aparna Areti^1^, Prashanth Komirishetty^1^, Ambika Chandrasekhar^1^, Chu Cheng^2^, and Douglas W. Zochodne^1,2 *^

^1^Neuroscience and Mental Health Institute and Division of Neurology, Department of Medicine, University of Alberta, Edmonton, Alberta, Canada.^2^Department of Clinical Neurosciences, University of Calgary, Calgary, Alberta, Canada. **^3^**Dept. of Anatomy, Physiology, and Pharmacology and Cameco MS Neuroscience Research Centre, College of Medicine, University of Saskatchewan, Saskatoon, Saskatchewan, Canada.

**Supplementary Table: 1:** Primer sequences

| Name | Primer sequence |
| --- | --- |
| Atg5 | Forward - 5’ AAC GAG AAG CAG AGC CAT AC 3’  Reverse - 5’ GGG TGT GCC TTC ATA TTC AAA C 3’ |
| LC3a | Forward - 5’ GCA CAG CAT GGT GAG TGT AT 3’  Reverse - 5’GAA GGT TTC TTG GGA GGC ATA G 3’ |
| Beclin1 | Forward - 5’ CAG GAA CTC ACA GCT CCA TTA C 3’  Reverse - 5’ CCA TCC TGG CGA GTT TCA ATA 3’ |
| RPLP | Forward - 5’ TACCTGCTCAGAACACCGGTCT 3’  Reverse - 5’GCACATCGCTCAGGATTTCAA 3’ |

**Supplementary Video 1 legend:** Time-lapse confocal images of live cultures of primary sensory neurons grown on PLL coated, but laminin deprived surfaces show macrovesicular formation.
