# Supplementary material for: Survival of compromised adult sensory neurons involves macrovesicular formation
Source: Cell Death Discov. 2022 Nov 24;8:462. doi: 10.1038/s41420-022-01247-3 (PMC9691713; doi:10.1038/s41420-022-01247-3)
Supplement: Supplementary file 6 — Supplementary Video 2 legend [file 41420_2022_1247_MOESM6_ESM.docx]

**Video 2:** Time-lapse confocal images of live cultures of primary sensory neurons grown on PLL coated, but laminin deprived surfaces show unstable neurites emerging from the macrovesicular structures followed by rearrangement of neuronal cytoskeleton.
